# Tangram: a comprehensive toolbox for mobile element insertion detection

**DOI:** 10.1186/1471-2164-15-795

**Published:** 2014-09-16

**Authors:** Jiantao Wu, Wan-Ping Lee, Alistair Ward, Jerilyn A Walker, Miriam K Konkel, Mark A Batzer, Gabor T Marth

**Affiliations:** Department of Biology, Boston College, Chestnut Hill, MA USA; Department of Biological Sciences, Louisiana State University, Baton Rouge, LA USA; Department of Human Genetics and USTAR Center for Genetic Discovery, University of Utah, Salt Lake City, Utah USA

**Keywords:** Structural variation, Mobile element insertion, Retrotransposon, Endogenous retrovirus, L1, Alu, SVA, High-throughput sequencing

## Abstract

**Background:**

Mobile elements (MEs) constitute greater than 50% of the human genome as a result of repeated insertion events during human genome evolution. Although most of these elements are now fixed in the population, some MEs, including ALU, L1, SVA and HERV-K elements, are still actively duplicating. Mobile element insertions (MEIs) have been associated with human genetic disorders, including Crohn’s disease, hemophilia, and various types of cancer, motivating the need for accurate MEI detection methods. To comprehensively identify and accurately characterize these variants in whole genome next-generation sequencing (NGS) data, a computationally efficient detection and genotyping method is required. Current computational tools are unable to call MEI polymorphisms with sufficiently high sensitivity and specificity, or call individual genotypes with sufficiently high accuracy.

**Results:**

Here we report Tangram, a computationally efficient MEI detection program that integrates read-pair (RP) and split-read (SR) mapping signals to detect MEI events. By utilizing SR mapping in its primary detection module, a feature unique to this software, Tangram is able to pinpoint MEI breakpoints with single-nucleotide precision. To understand the role of MEI events in disease, it is essential to produce accurate individual genotypes in clinical samples. Tangram is able to determine sample genotypes with very high accuracy. Using simulations and experimental datasets, we demonstrate that Tangram has superior sensitivity, specificity, breakpoint resolution and genotyping accuracy, when compared to other, recently developed MEI detection methods.

**Conclusions:**

Tangram serves as the primary MEI detection tool in the 1000 Genomes Project, and is implemented as a highly portable, memory-efficient, easy-to-use C++ computer program, built under an open-source development model.

## Background

Structural variations (SVs), like single nucleotide polymorphisms (SNPs), are a ubiquitous feature of genomic sequences and are major contributors to human genetic diversity and disease [1–3]. With the advent of next-generation sequencing (NGS) technologies providing vast throughput for individual resequencing, a number of new algorithms have been developed for various SV types, including copy number variations (CNVs) [4–8], and large deletion events [9]. These algorithms take advantage of various signals provided by NGS mapping algorithms, primarily read-depth (RD), and read-pair (RP) mapping positions. However, the computational identification of mobile element insertions (MEIs) with NGS data is less well established because mobile elements (MEs) are highly repetitive DNA sequences that are difficult to align against a reference genome with commonly used mapping strategies.

The most recent estimates indicate that more than half of the human genome is comprised of MEs [10]. Based on their propagation mechanisms, MEs can be divided into two classes. Class I elements encompass retrotransposons that move within a genome through a two stage copy process utilzing an RNA intermediate. In contrast, DNA transposons rely for their mobilization on a ‘cut and paste’ mechanism and are considered Class II elements [11–15]. While DNA transposons are thought to have largely ceased activity in primates about 37 million years ago [16], retrotransposons have continued to propagate throughout primate evolution including the lineage leading to humans [13, 17].

Retrotransposons represent the most successful MEs in primates and are major drivers of genome expansion in primates. They can be further subdivided based on the presence/absence of long terminal repeats (LTRs). In humans, the currently propagating non-LTR elements include the autonomous long interspersed element 1 (LINE1 or L1), and the non-autonomous Alu and SVA elements [12, 18]. While L1 and Alu elements have been active throughout primate evolution, SVA elements are hominid-specific [19]. Endogenous retroviruses (ERVs) belong to the family of LTR elements and have played a minor role in recent human evolution. In contrast, non-LTR elements have continued to propagate in the human lineage since the divergence from the lineage leading to chimpanzee. In fact, there is evidence for a recent increase in non-LTR expansion in the human lineage compared to chimpanzee [20]. Altogether, ME mobilization rates varied considerably throughout primate evolution with episodes of lower and higher expansion [13, 17]. Compared to a peak in Alu and L1 expansion in anthropoid primates about 35–40 million years ago [21, 22], the current insertion/duplication rate of these elements is substantially reduced. However, many genetic disorders, such as Crohn’s disease [23], hemophilia [14] and some cancers [24, 25], have been reported to be associated with their transposition activities.

To address effective detection of MEI events we developed an MEI detection pipeline around our SPANNER SV discovery tool (C Stewart, https://github.com/chipstewart/Spanner), and deployed it on the Pilot data of the 1000 Genomes Project [26]. Using this pipeline we compiled the most comprehensive catalog of MEI events in the human genome to date [27]. Although an effective SV detector used extensively in the 1000GP [28], SPANNER only uses RP signal, limiting the precision of breakpoint resolution, detection sensitivity, as well as the genotype accuracy that can be achieved. Also, although the pipeline that was built around SPANNER was able to utilize the SR signal, its split alignment algorithm is only compatible with reads collected using the now defunct 454 sequencing technology. This issue significantly restricts its detection capability to new sequencing data.

More recently, three NGS-based MEI detectors, RetroSeq [29], TEA [25] and VariationHunter [30], have been published, each with specific limitations. For example, TEA and VariationHunter do not report sample genotypes, limiting their use for single-sample detection pipelines e.g. in personal genome sequencing projects; or genotype data likelihoods that are essential for phasing structural variants together with SNPs and short INDELs. Also, none of these detectors efficiently integrate the SR and RP signals: VariationHunter detects MEIs using RP signal alone; RetroSeq and TEA only trigger SR analysis when RP signal suggests a potential MEI, and therefore miss events for which only SR evidence is available from the reads. Because of the steady increase in the read lengths generated by today’s sequencing technologies, there is a significant increase in the confidence of alignments spanning SV event breakpoints. Therefore, it is reasonable to expect that using both SR and RP signal on an equal footing, as primary observations for “nucleating” SV event calls, will lead to more sensitive detection than RP signal alone, or RP signal in combination with a secondary SR search. As a more practical point, the TEA and VariationHunter programs produce reports in non-standard formats, rather than the well established standard variant call format (VCF) [31], an issue for data communication and downstream analysis. Finally, all the above tools focus on the detection of non-LTR events, such as Alu, L1 and SVA events, and they do not address the detection of LTR elements in the human genome.

## Results and discussion

Here we report a fast and convenient MEI detection toolbox, Tangram, which effectively integrates signals generated by both RP and SR mapping. What sets our approach apart from existing methods is the “global” use of SR mapping: we perform an SR mapping step for all orphaned or substantially soft-clipped reads before the detection begins, and therefore both RP and SR mappings are available at the outset, and can nucleate SV event calls. We target both non-LTR and LTR ME types. The global use of SR mapping substantially improves the accuracy of identifying SV event boundaries (breakpoints) and our method produces sample genotypes as well as genotype likelihoods. Unlike other SV detection tools, Tangram is able to simultaneously process multiple sequence alignment (BAM) [32] files to call MEI events on population-scale data, and can deal with multiple fragment length libraries and a mixture of read lengths within a single detection step. Tangram is memory and central-processing-unit (CPU) efficient as analysis is carried out locally, i.e. event detection in any given region only requires reading the alignments within that region. To our knowledge, there are currently no other detectors that can provide such a comprehensive set of features required for the full characterization of MEIs within a single sample, or a large collection of samples.

### Performance evaluation on simulated datasets

We evaluated the detection and genotyping performance of Tangram with a series of *in silico* experiments involving the insertion of 1,000 full-length AluY and 1,000 5′ truncated L1 elements into the sequence of human chromosome 20, and generated simulated paired-end sequencing reads of various lengths with realistic base error properties (see Methods). After aligning these reads to the human reference genome sequence using our MOSAIK read mapping program [33], we used Tangram to detect MEI events and to generate sample genotype calls (see Tables 1 and 2). For comparison, we also ran the RetroSeq program (See Methods for the command line used to call MEIs) on the same dataset (aligned with the BWA mapping program, using default parameters, as instructed by the RetroSeq documentation), and compared detection sensitivity and genotyping accuracy for various read lengths and levels of sequence coverage, considering both heterozygous and homozygous events, i.e. cases where the MEI event is present in one or both chromosome copies within the cell. TEA and VariationHunter do not report sample genotypes, and therefore were not used in the comparisons.

**Table 1:** **MEI detection sensitivity for Alu elements**

			Tangram					RetroSeq
Ploidy	Read length	Coverage	Sen (RP)	Sen (SR)	Sen (RP\SR)	Sen (SR\RP)	Sen (Union)	Sensitivity
Het	76 bp	5×	67.6%	60.0%	25.4%	17.8%	**85.4%**	43.7%
		10×	83.4%	88.9%	8.8%	14.3%	**97.7%**	93.6%
		20×	84.2%	97.8%	1.2%	14.8%	**99.0%**	98.9%
	106 bp	5×	45.1%	67.3%	13.9%	36.1%	**81.2%**	12.0%
		10×	77.0%	93.0%	4.5%	20.5%	**97.5%**	68.9%
		20×	83.4%	98.9%	0.4%	15.9%	**99.3%**	97.7%
Homo	76 bp	5×	83.4%	88.9%	8.8%	14.3%	**97.7%**	95.2%
		10×	84.2%	97.8%	1.2%	14.8%	**99.0%**	98.8%
		20×	84.6%	99.1%	0.4%	14.9%	**99.5%**	99.2%
	106 bp	5×	77.0%	93.0%	4.5%	20.5%	**97.5%**	68.9%
		10×	83.4%	98.9%	0.4%	15.9%	**99.3%**	97.7%
		20×	83.8%	99.3%	0.4%	15.9%	**99.7%**	98.9%

Results are shown for the Tangram and RetroSeq programs applied to simulated data (1,000 AluY insertions introduced at random positions on human chromosome 20). Simulated reads were generated under different ploidy values (homozygous or heterozygous), read length (76 bp and 106 bp) and read coverage (5X, 10X, 20X). Columns “Sen (RP)” and “Sen (SR)” indicate the sensitivity of the RP and SR methods respectively. The two columns “Sen (RP\SR)” and “Sen (SR\RP)” indicate the sensitivity of the RP and SR signal in isolation respectively. “Sen (Union)” indicates the overall sensitivity of Tangram when calling MEI with both RP and SR modules. The best result in each row is indicated in boldface text.

**Table 2:** **Genotype accuracy results of MEI detection using Tangram and RetroSeq on simulated data for Alus**

		Tangram			RetroSeq		
Read length	Coverage	Het	Homo	Total	Het	Homo	Total
76 bp	5×	99.3%	90.8%	97.6%	2.3%	92.8%	20.4%
	10×	100.0%	94.2%	98.8%	40.6%	63.6%	45.2%
	20×	100.0%	98.4%	99.7%	96.5%	8.8%	78.9%
106 bp	5×	96.6%	93.4%	96.0%	0.0%	91.6%	18.3%
	10×	99.6%	92.6%	98.2%	38.8%	64.4%	43.9%
	20×	100.0%	95.6%	99.1%	95.1%	10.8%	19.6%

As Table 1 shows for Alu detection, Tangram’s sensitivity exceeds 97% both for heterozygous and homozygous events in 10X sequence coverage or greater. Even in low-coverage sequence (5X is the approximate average sequence coverage in the low-coverage 1000GP datasets), Tangram maintains >80% sensitivity. Tangram’s sensitivity substantially exceeds that of the RetroSeq program, especially when detecting heterozygous events in low-coverage (5X) data. Tangram also boasts high specificity, making no false positive calls in any of the simulated data. This was also the case for RetroSeq.

We also tabulated genotype-calling accuracy, i.e. the rate at which a given algorithm provides the correct genotype for a given simulated sample (i.e. no MEI, heterozygous MEI, homozygous MEI). As Table 2 indicates for Alu detection, Tangram is able to call sample genotypes with >90% accuracy for all coverage levels and event ploidy we considered. Accuracy in our simulated data is nearly perfect for heterozygous events over 10X coverage, and for homozygous events over 20X coverage. These accuracy values compare very favorably with those obtained for RetroSeq, which appears to heavily favor homozygous calls in low-coverage data, and heterozygous calls in deeper sequence coverage and has a very high error rate in the non-favored category. The overall accuracy of the Tangram genotypes, obtained by a judicious weighting of heterozygous and homozygous events, is high, over 96%, in every category, again, substantially higher than what was obtained with RetroSeq.

**Figure 1:**
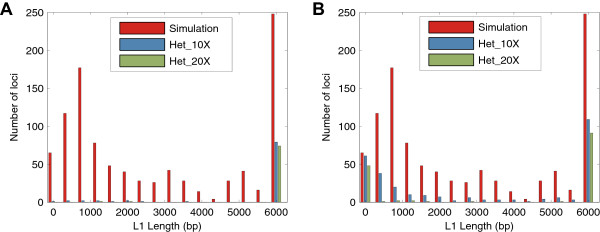
**L1 length distributions of missed events. A** and **B** show the length distributions of L1 events that are not detected by Tangram and RetroSeq, respectively. The red line is the L1 length distribution of the 1,000 L1 elements introduced in the simulated data. The blue line represents the missed events in 10X data and the green line represents the missed events in 20X data. Tangram **(A)** detected almost all the severely truncated events whereas RetroSeq **(B)** is not sensitive enough to those short L1 elements.

L1 elements in the human genome are usually found truncated at the 5′ end [34], which further complicates detection. To assess the sensitivity of our method to those truncated L1 elements (L1 Homo sapiens, L1HS), we generated two simulated datasets using the same strategy as the Alu simulations with 5′ truncated L1 elements (See Methods); heterozygous 106 bp at 10X and 20X sequence coverage. The length distribution we used was derived from the L1 detection results in Stewart et al. 2011. The results are shown in Table 3. For both datasets, Tangram achieved over 90% sensitivity and genotype accuracy, which is substantially better than the performance of RetroSeq. Moreover, from Figure 1A and 1B we can see that Tangram can effectively detect those severely truncated L1 events whereas RetroSeq missed almost all the short L1 elements (<150 bp). Like the Alu simulation dataset, both detectors do not report any false positive L1 events.

**Figure 2:**
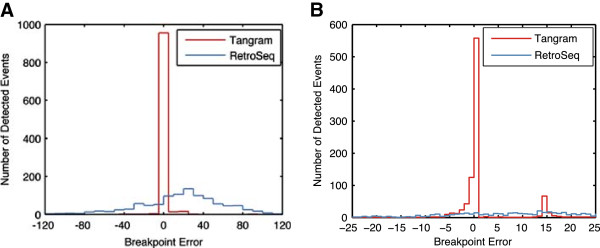
**Breakpoint resolution for A (AluY) and B (L1).** The difference between reported and true breakpoint position in simulated data is shown for the Tangram and the RetroSeq MEI detection algorithms (homozygous events in 106 bp paired-end reads, 20X sequence coverage for AluY simulation and heterozygous events in 106 bp paired end reads, 20X sequence coverage for L1 simulation). The majority of breakpoints reported by Tangram exactly match the true breakpoint.

Determining the exact location of SV event boundaries is notoriously difficult. In the simulation experiments performed here, Tangram was able to assign MEI breakpoints at or near single nucleotide resolution using the SR signal. For Alu detection with 106 bp reads at 20X (homozygous), greater than 65% of the reported breakpoints co-locate exactly with, and over 99% are within 15 bp of the true breakpoints (see Figure 2A). For L1 detection with 106 bp reads at 20X (heterozygous), more than 60% of the reported breakpoints co-locate exactly with, and over 97% are within 15 bp of the true breakpoints (see Figure 2B). The inexactness is caused by the simulated target site duplication (TSD) sequences (See Methods). This introduces a localization error mode. Additional, smaller localization errors are caused by alignment artifacts where similarity exists between the TSD and the ME sequences themselves. This performance is attributable to SR-mapped reads identifying the breakpoints at a resolution that RP-only methods are unable to match. See Methods for detailed information about breakpoint calculation.

**Table 3:** **MEI detection sensitivity and genotype accuracy for L1 elements**

	Tangram		Retroseq	
	Sen	Genotype	Sen	Genotype
Het_106bp_10X	**90.9%**	**92.2%**	71.5%	27.0%
Het_106bp_20X	**92.4%**	**97.7%**	85.3%	90.6%

Results are shown for the Tangram and RetroSeq programs applied to simulated data (1,000 L1 insertions randomly truncated at the 5′ end at random positions in human chromosome 20). “Sen” indicates sensitivity and “Genotype” indicates the genotype accuracy. The best result in each row is indicated in boldface text.

### Performance comparisons using 1000 genomes project data

We ran Tangram and two other MEI detection algorithms, RetroSeq and TEA, to analyze deep-coverage sequencing data from a CEU trio consisting of samples NA12878 (89X), NA12891 (78X) and NA12892 (78X), obtained from the public 1000GP ftp site (the DNA for 1000GP sequencing analysis is sampled from blood cells). The data consists of 101 bp paired-end reads generated by Illumina HiSeq sequencing machines; the insert size was 465 ± 50 bp (median ± standard deviation). We mapped the reads with MOSAIK 2.0 for Tangram and BWA for RetroSeq and TEA, according to author instructions. To assess sensitivity and genotype accuracy, we compared the MEI loci (Alu and L1) reported by the three detectors to the events reported and experimentally characterized in a previous large-scale study using an earlier set of 1000GP data [27] for the same samples (characteristics of this dataset from the 1000GP Pilot 2 trio data are reported in Table 4). The Stewart et al. call set consisted of 1,208 Alu and 180 L1 calls, including 486 Alu and 48 L1 insertions that were experimentally confirmed with a polymerase-chain-reaction-based (PCR-based) validation techniques. As shown in Table 5, Tangram recovered >98% of PCR validated events and >93% of all reported events. RetroSeq provided comparable results, but TEA was unable to achieve this level of sensitivity to Alu events. Tangram’s genotype accuracy for Alu events was >91% for all three samples. Tangram detected approximately 87% of PCR validated L1 insertion events, outperforming the two competing algorithms. Tangram’s sensitivity to L1 events reported in the Stewart et al. data set drops markedly in comparison to the PCR-validated events. This is likely the result of the high false discovery rate (FDR) for L1 events (18.8%) in the Stewart et. al. data set. Notably, our algorithms called none of the events reported in the Stewart et al. dataset that failed PCR validation. It is noteworthy that sample NA12878 had the highest number of MEI calls using either of the calling methods. This is likely the result of the substantially higher read coverage in this sample, as well as longer reads from 454 sequencing machines, not available for the other two samples in the analysis of Stewart et al. 2011 (Table 4).

**Table 4:** **Sequence coverage for two sequencing technologies of CEU trio used in 1000GP Pilot MEI paper**

ID	454	Illumina
NA12878	11.0X	15.9X
NA12891	0.0X	14.9X
NA12892	0.0X	9.2X

**Table 5:** **Sensitivity and genotype accuracy in deep coverage sequencing data from the 1000 Genomes Project**

		Stewart et al. 2011		Tangram			RetroSeq			TEA		
	Sample	MEI loci		Sensitivity		Genotype	Sensitivity		Genotype	Sensitivity		Genotype
		Validated	Reported	Validated	Reported		Validated	Reported		Validated	Reported	
Alu	NA12878	408	965	**98.8%**	**93.0%**	**95.0%**	94.1%	87.7%	76.4%	89.5%	82.2%	N/A
	NA12891	309	675	98.1%	96.3%	**91.2%**	**98.4%**	**96.4%**	67.9%	96.1%	93.8%	N/A
	NA12892	312	650	98.1%	96.9%	**92.6%**	**99.0%**	**97.4%**	71.2%	94.2%	92.5%	N/A
L1	NA12878	38	157	**86.8%**	**52.2%**	**87.5%**	78.9%	45.8%	83.3%	84.2%	49.7%	N/A
	NA12891	26	64	**92.3%**	**75.0%**	**100.0%**	76.9%	64.1%	66.7%	84.6%	70.3%	N/A
	NA12892	34	76	**94.1%**	**76.3%**	**85.7%**	79.4%	65.8%	50.0%	76.5%	64.5%	N/A

Comparisons are shown for a CEU trio (NA12878, NA12891 and NA12892) processed with Tangram, RetroSeq and TEA. Sensitivity and genotype accuracy was measured by comparing the reported events with those in Stewart et al., 2011. The total number of validated and reported MEI loci are shown under the “Stewart et al. 2011” column. The two sub columns under each detector, “Validated” and “Reported”, show the sensitivity to PCR validated loci and all reported loci in Stewart et al. 2011, respectively. The TEA program does not provide genotype calls, and therefore could not be used for genotype accuracy comparisons. The best result in each row is indicated in boldface text.

Our experiments here demonstrate that Tangram provides accurate MEI genotypes across all MEI types (see Table 6). The TEA program does not provide sample genotypes, and therefore was not included in this comparison. RetroSeq appears to suffer from a systematic bias when applied to deep-coverage data; it called almost all MEI loci as heterozygous. In comparison, Tangram can effectively distinguish between homozygous and heterozygous loci.

**Table 6:** **Genotype accuracy**

			Tangram		RetroSeq	
		Genotype from validation	Genotype call		Genotype call	
			Het	Homo	Het	Homo
Alu	NA12878	Het	120	8	119	0
		Homo	1	26	37	1
	NA12891	Het	95	13	93	0
		Homo	0	40	44	0
	NA12892	Het	106	11	104	0
		Homo	0	32	42	0
L1	NA12878	Het	5	1	4	0
		Homo	0	2	1	1
	NA12891	Het	4	0	2	0
		Homo	0	2	1	0
	NA12892	Het	3	1	3	0
		Homo	0	3	3	0

A contingency table is shown for MEI genotypes reported by Tangram and RetroSeq on deep coverage sequencing data from a CEU trio (NA12878, NA12891 and NA12892). The “Genotype from validation” column shows the genotype that was validated in Stewart et al. 2011. The “Genotype call” column shows the genotype predicted by Tangram and RetroSeq at the same loci. The “Genotype” column in Table 5 was calculated based on the results in this table.

### Running Tangram on population data

**Figure 3:**
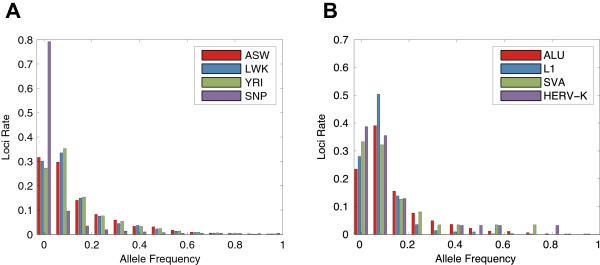
**Allele frequency spectrum of African populations.** The number of loci for a given allele frequency is normalized with the total number of loci. **A**. Allele frequency spectrum for MEI and SNP (provided by Sanger Institute with QCall and GATK, chromosome 20 only) variants detected in 3 African populations. Results for samples designated as ASW, LWK and YRI are shown, for 4 types of MEIs: Alu, L1, SVA and HERV-K. Overall, the AFS of MEIs is very similar to that of SNPs except that there is limited sensitivity to low frequency events for MEI detection due to sparse or absent supporting reads in low-coverage data. **B**. Allele frequency spectrum for four different types of MEs, Alu, L1, SVA and HERV-K detected with the Tangram toolbox.

We deployed Tangram on 218 samples from the 1000GP Phase 1 release [35]. Three populations were included in this dataset: African ancestry in Southwest USA (ASW, 50 individuals), Luhya in Webuye, Kenya (LWK 83 individuals) and Yoruba in Ibadan, Nigeria (YRI, 85 individuals). On average, each sample had 5X sequence coverage so the overall coverage of this dataset is ~1,000X. The allele frequency spectrum (AFS) of all MEIs for each of the three populations (4,085 Alu, 1,548 L1, 88 SVA and 44 HERV-K insertions) and AFS of SNP calls generated by Sanger Institute with QCall [36] and GATK [37] on the same sequencing dataset (chromosome 20 only) are shown in Figure 3. The expectation is that the AFS of MEIs is similar to the AFS observed for SNP data [27]. This is indeed the case (Figure 3A), except at low allele frequency, where detection sensitivity drops off in the low-coverage 1000GP datasets (as there may be too few RP and/or SR mapped reads supporting an MEI event). Additionally, we calculated the allele frequency spectrum for each ME type. Figure 3B shows the AFS of four ME types, Alu, L1, SVA and HERV-K, across all three populations. Similarly, we can see from the figure that Tangram loses some sensitivity on low allele-frequency events.

### Experimental validation

To assess the specificity of Tangram, we performed PCR validation on 23 1000GP Phase 1 [35] samples (Table 7), including a CEU trio (NA12878, NA12891 and NA12892) with deep coverage (~50X) and 20 low-coverage (~5X) samples from the CHS and LWK populations (the DNA for the PCR validation experiment was sampled from the corresponding cell line). Tangram detected 2,874 Alu, 256 L1, 53 SVA and 22 HERV-K insertions in these samples. Of the 3,205 loci, 357 were novel, i.e. not reported in previous studies [27, 38–44], and absent from the dbRIP database [45]. Two random subsets, 160 sites in all, were randomly selected for PCR validation: (1) 80 loci (66 known + 14 novel) were randomly selected from the entire call set of 3,205 MEIs; and (2) additional 80 loci were randomly selected only from the 357 novel calls. PCR validation results for Tangram and VariationHunter are shown in Table 8 and Table 9. Tangram achieved very low FDR for all three non-LTR MEI types (<6%). Although the numbers are low, no false positive L1 and SVA calls were reported. The overall estimated FDR for the first and second validation sets were 2.53% and 9.21%, respectively. This result is consistent with expectations that newly detected, previously unknown events have higher FDR. In Table 9, we compared experimental validation results for three algorithms: Tangram, RetroSeq, and VariationHunter, for event types detected by each calling algorithm. Tangram achieves substantially higher specificity than the two competing algorithms. In fact, this level of accuracy is close to the FDR of SNP calls from current state-of-the-art variant callers [35].

**Table 7:** **Sequencing information of CEU trio and 20 1000GP phase I samples used for PCR validation**

Sample	Population	Platform	Coverage	Read length
NA19397	LWK	ILLUMINA	5.9X	101 bp
NA19398	LWK	ILLUMINA	5.6X	101 bp
NA19399	LWK	ILLUMINA	5.5X	101 bp
NA19404	LWK	ILLUMINA	5.8X	101 bp
NA19428	LWK	ILLUMINA	6.2X	101 bp
NA19429	LWK	ILLUMINA	6.6X	108 bp
NA19434	LWK	ILLUMINA	5.6X	108 bp
NA19435	LWK	ILLUMINA	5.9X	108 bp
NA19440	LWK	ILLUMINA	16.9X	108 bp
NA19443	LWK	ILLUMINA	12.4X	108 bp
HG00662	CHS	ILLUMINAHiSEQ	5.2X	91 bp
HG00663	CHS	ILLUMINAHiSEQ	5.7X	91 bp
HG00671	CHS	ILLUMINAHiSEQ	5.9X	91 bp
HG00672	CHS	ILLUMINAHiSEQ	5.6X	91 bp
HG00683	CHS	ILLUMINAHiSEQ	5.4X	91 bp
HG00684	CHS	ILLUMINAHiSEQ	5.8X	91 bp
HG00689	CHS	ILLUMINAHiSEQ	5.4X	91 bp
HG00690	CHS	ILLUMINAHiSEQ	5.8X	91 bp
HG00464	CHS	ILLUMINAHiSEQ	1.3X	91 bp
HG00614	CHS	ILLUMINAHiSEQ	4.6X	91 bp
NA12878	CEU	Multiple	65.3X	47 bp ± 15 bp
NA12892	CEU	Multiple	47.3X	46 bp ± 10 bp
NA12891	CEU	Multiple	43.0X	45 bp ± 12 bp

The sequencing data for CEU trio is a mixture of multiple libraries with different read length so in the “Read length” column for these three samples shows mean ± standard deviation.

**Table 8:** **PCR validation results for the Tangram MEI detector**

	ALU		L1		SVA		HERV-K		Total	
	Random	Novel	Random	Novel	Random	Novel	Random	Novel	Random	Novel
Analyzed by PCR	68	64	7	3	3	6	1	3	80	78
Validated Loci	66	58	7	3	3	6	1	2	77	69
Invalidated Loci	2	6	0	0	0	0	0	1	2	7
FDR	2.94%	9.38%	0.00%	0.00%	0.00%	0.00%	0.00%	33.33%	2.53%	9.21%

Validation results and estimated false discovery rates are shown for MEI calls from 23 1000 Genomes Project Phase 1 samples.

**Table 9:** **Comparison of PCR validation results across three MEI detection algorithms**

	Tangram			RetroSeq			VariationHunter		
	Random	Novel	Combined	Random	Novel	Combined	Random	Novel	Combined
Analyzed by PCR	80	78	158	80	80	159	83	51	134
Validated Loci	77	69	142	73	58	131	69	29	98
Invalidated Loci	2	7	9	7	21	28	14	22	36
FDR	2.53%	9.21%	5.96%	8.75%	26.58%	17.61%	16.87%	43.14%	26.86%

Calls were made in 23 1000 Genomes Project Phase 1 samples by Tangram, RetroSeq and VariationHunter.

### Resource requirements and software availability

The primary motivation behind developing Tangram was to provide highly accurate MEI calls. To be a useful software tool, however, it must be easy to install, easy to run, and able to generate results in a timely fashion, using reasonable computational resources. We characterize resource usage and analysis time on our analysis of the 218 1000GP low-coverage samples (the average coverage is about 5X) [35]. When using other MEI detection software programs, it is a common requirement that only a single BAM file can be processed at a time, necessitating all input BAM files to be merged into a single file (a lengthy task), or to process each BAM file individually (reducing sensitivity to low-frequency events). Tangram, in contrast, can process all input BAM files simultaneously. Most currently available structural variant callers employ multiple passes through the entire input file, requiring substantial memory and computation time. To reduce the memory footprint and increase the throughput, Tangram was designed to call MEI events regionally, i.e. within shorter windows of the sequence alignment. Single-pass analysis is made possible by annotation tags produced by our MOSAIK read mapper software, marking reads whose fragment-end paired mate maps into ME reference sequence. Additional parallelization was accomplished by multi-threaded implementation of the software. In this test, we submitted one Tangram detection job for each chromosome (chromosome 1 - chromosome X). Each job used one AMD Opteron 6134 CPU (8 cores at 2.3 GHz). The detection process finished within 58 hours (wall time) or 96 hours (CPU time). Tangram is designed to run on any specified genomic region, e.g. chr1:10,000-20,000, to facilitate parallelization when a computer cluster is available for running the analysis. For example, when we repeated the detection process in 1Mbp detection windows running in parallel on our cluster, the total compute only took 0.24 hours (wall time) or 0.40 hours (CPU time).

As inherent to its algorithmic design, Tangram requires mappings to ME reference sequences, as well as BAM alignment file tags that are currently only provided by our own MOSAIK mapper. As discussed below, we are developing and testing a program to “retrofit” alignments created with other read mapping programs such as BWA or BOWTIE [46], to provide similar information as part of an alignment post-processing step, to enable efficient MEI detection using the primary mappings. But for now, before we are able to release this post-processor, we recommend remapping with MOSAIK. MOSAIK is a fast read mapper, able to map over 80 read pairs (100 bp Illumina) per second [33].

Tangram is easy to install and run. Users can download it from its main github repository (https://github.com/jiantao/Tangram). We have also integrated it into our pipeline and tool launcher system, GKNO, available at http://gkno.me.

## Conclusions

MEI events can have a strong impact on gene function and therefore accurate detection and genotyping is essential within individuals. MEs are, by nature, repetitive sequences and are therefore difficult to detect. To our knowledge, our Tangram software is the only robust software tool capable of detecting all classes of MEIs, providing accurate individual genotype information, and facilitating near base-perfect breakpoint localization. We showed that Tangram could achieve higher sensitivity, specificity, genotyping accuracy, and breakpoint accuracy than competing MEI detection methods because of the global use of SR mapping information into the detection process. Competing algorithms either only use RP mapping information to call events, or perform SR mapping in regions where RP mappings indicate a possible MEI event. In contrast, Tangram analyzes both RP and SR mapped reads from the start, and can therefore detect events for which only SR mapping evidence exists.

Table 1 illustrates detection sensitivity when RP or SR signal is used in isolation, or in combination with each other. At almost all read lengths and coverage values, the SR method on its own is more sensitive than the RP method (except for low, 5X coverage in 76 bp reads). Importantly, RP detection sensitivity does not exceed 85%, even in deep-coverage data. This is because RP-mapped reads localize the MEI point to a window. If the reference sequence already contains an ME within this window, one must filter out the candidate event because of the high likelihood of spurious detection. SR mapping localizes the insertion site with much greater resolution, making it possible to distinguish between MEs in the reference, and polymorphic insertions not present in the reference.

Table 1 also illustrates that RP-based methods implementing a secondary SR-mapping step can perform very well in deep-sequencing data because such high-coverage datasets likely contain RPs that map across the breakpoints and additional reads that can be SR-mapped across the breakpoint for fine localization. In low-coverage data however, there are many events without RPs mapping across the breakpoints. When using shorter reads, reliable SR mapping becomes difficult. In both cases, sensitivity suffers. Recent technological developments are continuously increasing the length of sequenced reads. Consequently, the same sequence coverage is accomplished with fewer, but longer, reads. Moving forward, this trend clearly favors the SR mapping method, and in particular, methods that use SR mapping as part of their primary detection approach. As we demonstrated in this study, such methods are more sensitive and specific, have higher genotype accuracy, and are able to localize event boundaries more accurately. Admittedly, our sensitivity estimates are likely too high, because our method is not designed to detect MEIs that are embedded inside other MEs in the genome. However, these estimates are perfectly valid for comparing the performance of Tangram to that of competing tools. A clear limitation of our method is that it only detects MEIs for which a ME reference sequence is provided in the mapping step. The detection of “novel” insertions is a much bigger, and as of today a largely unsolved problem.

Our main focus in this study was on Alu insertions, and the balance of simulated datasets we used to characterized our method reflects this. Biologically, Alus are the most abundant MEIs in the human genome. Methodologically, the majority of competing approaches also focus on Alu (and in some cases, ONLY on Alus). However, Tangram is also able to effectively detect L1 insertions, as demonstrated both with simulations and with the analysis of real datasets.

As mentioned earlier, currently Tangram can only run on alignment data generated by the MOSAIK aligner, but not by other widely used sequencing aligners such as BWA, because only MOSAIK currently provides the mapping information vital for MEI detection with our method. We realize that it would be desirable to run Tangram on e.g. BWA alignments, and have written a program, “tangram-bam” currently in testing, that is able to add to the primary BAM file the appropriate mapping information, at the cost of very light additional computation. With this modification Tangram will not only be compatible with MOSAIK and BWA but also with other primary read mapping programs.

## Methods

### The Tangram detector - algorithmic overview

**Figure 4:**
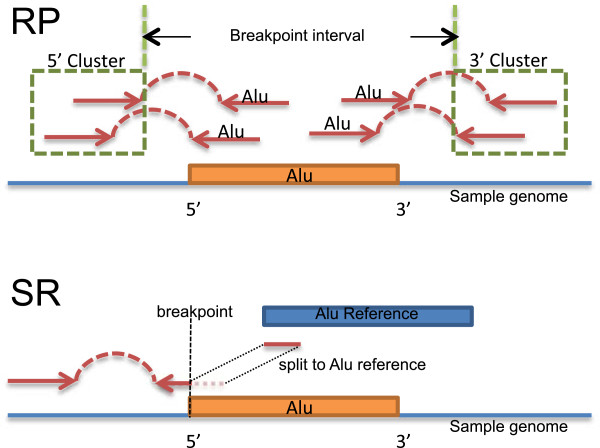
**Illustration of RP and SR detection methods.** The top panel demonstrates our RP MEI detection method. The blue line with orange box represents a sample genome with an MEI. Each pair of red arrows represent a read pair aligned to this genome. For RP method, we collect those repairs that one mate aligned uniquely to the genome the other mate aligned into the inserted ME. By clustering those uniquely aligned mates (green dashed boxes) we can estimate the insertion location. The type of inserted element is provided by MOSAIK aligner (ZA tags). The breakpoint confidence interval is given by the distance between two uniquely aligned mates that are closest to the real breakpoint. For SR method, the bottom panel, we collect those read pairs that one mate is uniquely aligned to the genome and the other mate is either unaligned or soft-clipped. The unaligned or soft-clipped read will be split into two segments: one of them will be aligned back to the normal human genome reference and the other segment will be aligned to the ME reference (blue box). The breakpoint can be determined by the alignment location of the first segment.

As input, Tangram uses reads aligned to the genome reference sequence as well as to ME reference sequences obtained from RepBase [47], available in a customized BAM format alignment file(s) that contains MEI detection information within an optional field, the ZA tag, to indicate that a read’s mate (in the case of fragment-end read pairs) maps to one of the ME reference sequences. Currently, these special alignments to ME reference sequences can be produced by the MOSAIK mapping software during its primary aligning process (a specific command line argument has to be given to MOSAIK) [33] (version 2.0 or above). Tangram’s RP detection module first scans the alignment for read pairs where one mate uniquely aligns to the genome reference, and the other mate maps to a ME reference sequence. Secondly, read pairs where one mate is aligned to the genome reference uniquely (i.e. with high read mapping quality value, or MQ) and the other mate is either soft-clipped or unaligned, are collected as the starting material for SR mapping. The integrated SR sub-module in Tangram attempts to align these soft-clipped or unaligned mates both to the genome reference and to the ME reference sequences using the split read algorithm (i.e. aligning one section of the read to the reference genome and another section to the ME reference). Loci in the genome with either RP or SR evidence for a candidate MEI event are then extracted. An illustration of these two methods is shown in Figure 4. Candidate events are filtered on the number and type of supporting fragments. A genotyping module produces individual genotype likelihoods and calls sample genotypes. A reporting module produces a VCF format variant report including the location and type of the events, as well as individual sample genotype information. All three modules, RP, SR and genotyping are integrated in a single piece of software so there is no intermediate steps or output for detection.

### Sequence alignment to genome and mobile element reference sequences

Alignments were created with the MOSAIK program, a hash-based read mapper that is aware of user-specified insertion sequences, e.g. MEIs. When the insertion sequences are provided, the reference hashes are prioritized such that alignment to the MEI sequences are attempted prior to alignment to the genome reference. Since MEIs are repetitive elements, a read from an MEI can be mapped to several locations within the genome (potentially hundreds of locations). While MOSAIK aligning sequencing reads, an additional field inside the BAM file, the ZA tag, is then populated with information about the read mate, including MEI information, location, mapping quality and number of mapping locations for the mate. This information ensures that BAM search operations (which can be lengthy for large alignment files) can be avoided.

### MEI detection based on read-pair (RP) mapping positions

Tangram first establishes the fragment length distribution for each library in the input BAM files using ‘normal’ read pairs (i.e. those read pairs where both mates are uniquely aligned to the same chromosome with expected orientation). Tangram then searches the BAM files for MEI-candidate read pairs that have one mate uniquely aligned to the reference genome and the other aligned to a ME reference. Such read pairs must also satisfy one of the following three requirements: 1) they do not have the expected orientation; 2) they are not aligned to the same chromosome (not including the MEI references), or 3) the fragment length is not consistent with the fragment length distribution (p-value ≤ 0.005). For each type of ME (Alu, L1, SVA and HERV-K), Tangram clusters the uniquely aligned mates of these candidate read pairs with a customized nearest-neighbor algorithm [48, 49] according to their fragment center position (aligned position of the uniquely aligned mate plus one half of the median of the fragment length distribution). During this process read pairs cluster with other read pairs within a range determined by the fragment length distribution. This algorithm can handle candidate read pairs from different libraries and samples effectively, which can significantly improve the sensitivity for multiple low-coverage samples. Also, the complexity of this algorithm is linear in the number of candidate read pairs, making it suitable for large-scale sequencing data. Read pairs that span into MEs from the 5′ end will be clustered separately from those spanning in from the 3′ end. Tangram identifies an MEI event if a pair of clusters in the MEI neighborhood range span into the insertion from both the 5′ and 3′ ends. The true breakpoint should locate somewhere between the end of the 5′ cluster or the beginning of the 3′ cluster (Figure 4). Tangram reports the estimated breakpoint following a leftmost convention (smallest genomic coordinate of the two positions).

### MEI detection based on split-read (SR) mapping positions

**Figure 5:**
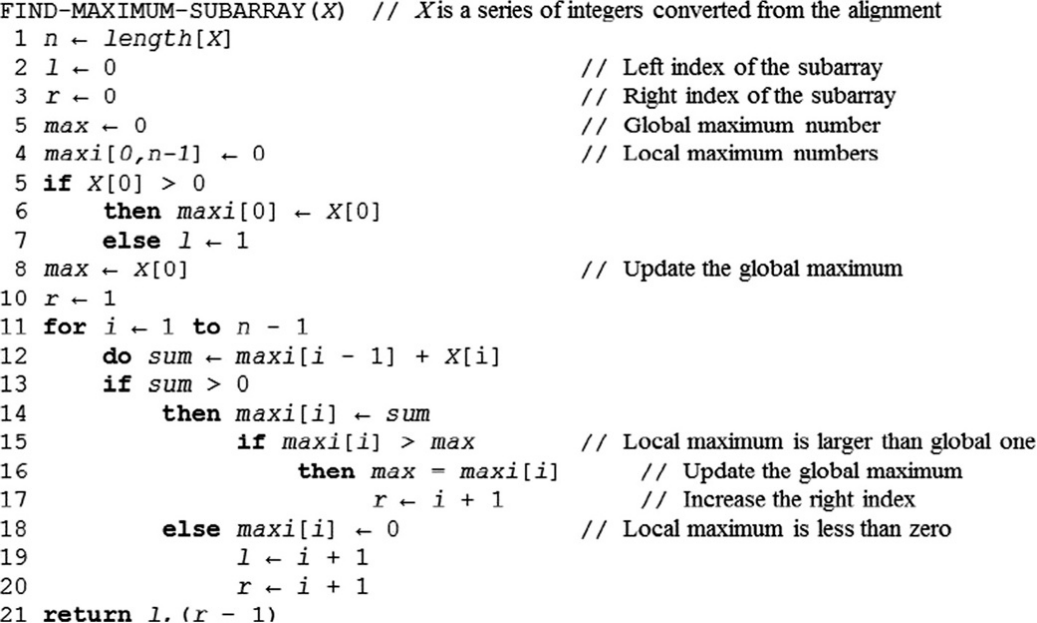
**Algorithm of finding maximum subarray that is used in SCISSORS.**

The Scissors (https://github.com/wanpinglee/scissors) split-read mapping package was integrated into our MEI detector as a library providing an application programming interface (API) to its functions. When mapping reads that span ME insertions, SCISSORS uses a sensitive and fast algorithm, *single instruction multiple data* Smith-Waterman (SIMD SW or SSW, https://github.com/mengyao/Complete-Striped-Smith-Waterman-Library), with match, mismatch, gap opening, and gap extending scores of 1, −3, −5, and −2 respectively, to obtain partial alignments against the reference genome (see the left partial alignment shown in the bottom panel of Figure 4). Then, SCISSORS attempts to map the read to known insertions. SCISSORS hashes and stores the known insertions in a hash table. For each read, SCISSORS uses these hashes to generate candidate alignments and finally applies the SSW to these candidates to obtain the second partial alignment against these insertion sequences (see the right partial alignment to the ME reference shown in the bottom panel of Figure 4). The sequences may be inserted on the reverse strand so SCISSORS also checks the reverse complement of the inserted sequences. Since the exact breakpoint in a read has not been determined before aligning, the entire read is necessary for aligning against either the local Smith-Waterman (SW) region or inserted sequences. The entire unmapped read is taken for mapping to the Smith-Waterman (SW) region. The read is also taken for mapping to inserted sequences. Hence, the tails of each partial alignment generated by SSW often contain mismatches with respect to the reference or inserted sequence. This is often seen at the SV breakpoints. SCISSORS attempts to clean up these regions by solving a maximum subarray problem (Figure 5). This problem was first proposed by Ulf Grenander in 1977. First, an alignment is converted into a one-dimensional array of numbers using the following scheme. Each base in the alignment is assigned the value +1 if the base matches the reference or −5 otherwise (mismatches and gaps). Then, Kadane’s algorithm [50] is used to determine the subarray with the largest sum in time complexity O(n). The resultant subarray indicates the best portion of the alignment that maps to the reference or the inserted sequence. This algorithm permits the use of a more lenient Smith-Waterman score, since eventually the junk portion of alignments (with respect to the reference genome or inserted sequences) will be trimmed off. Using a lenient Smith-Waterman score and this clean-up approach results in longer pairwise alignments (including longer gaps).

### Candidate MEI event filtering and post-processing

The MEI candidates are first filtered using the number of supporting fragments. An MEI candidate with at least two RP supporting fragments from both 5′ and 3′ or at least two SR supporting fragments were retained. Candidates that were supported by RP signal alone undergo additional filtering. If the candidate MEI falls within a predefined distance of a locus of the reference genome annotated by RepeatMasker [51] downloaded from UCSC Table Browser [52] they are removed from the candidate list. The distance used is the approximate maximum expected fragment length (p-value ≈ 0.005) in the clusters of supporting RP fragments. For Alu and HERV-K events, the candidate call is only filtered out if the MEI in RepeatMasker is also an Alu or HERV-K event. L1 and SVA elements are filtered out if they also co-locate with their corresponding referenced ME or Alu events in RepeatMasker. For MEI events supported by SR signal, no further filtering steps are applied. All remaining MEI candidates are reported in the final VCF file. These filtering steps can be performed using the PERL program (tangram_filter.pl) that is included in the toolbox.

### Sample genotype calling and genotype likelihood calculation

Tangram uses a Bayesian framework to predict the genotype of MEI events [27]. We calculate the posterior probability of a given sample MEI genotype *g* (i.e. monomorphic: REF/REF; heterozygous MEI: REF/MEI: or homozygous MEI: MEI/MEI) as follows:


where *D* is the observed read evidence at the site; and *P*(*g*) is the prior probability of the genotype. By default, Tangram sets a flat prior probability (1/3) for all three possible genotypes. The data likelihood, P(*D*|*g*), is calculated as a binomial probability with the following parameters:


where *p*_*g*_ is the expected ratio of MEI alleles to the total number of fragments (0 for homozygous reference, 0.5 for heterozygous MEI and 1 for homozygous MEI); *N*_*ref*_ and *N*_*alt*_ are the numbers of read-pair fragments that support reference and MEI (alternate) alleles, respectively. Reference and MEI alleles are defined as follows: any uniquely mapped read pairs spanning the predicted breakpoint with a consistent insert size and orientation will be counted as a fragment supporting the reference allele. Fragments supporting an alternate allele (insertion) are those inconsistent with the conditions for a reference allele collected during the detection step (both RP and SR signal). The meaning of the data likelihood is the binominal probability that *N*_*ref*_ + *N*_*alt*_ will fluctuate to *N*_*alt*_, given the expected *p*_*g*_.

The genotype reported by Tangram is that with the highest posterior probability and the output VCF file is populated with the corresponding data likelihoods.

### Simulation data generation

We evaluated the detection and genotyping performance of Tangram with a series of experiments using simulated data based on hg19 (human genome reference) chromosome 20. One thousand full-length AluY elements with a 15 bp poly-A tail and a 15 bp target-site duplication (TSD) sequence were randomly introduced into chromosome 20. No elements were allowed to insert within a 100 bp window of the reference MEs or other simulated elements. Simulated Illumina paired-end reads were generated for both heterozygous and homozygous insertions, with two different read lengths (76 bp and 106 bp) and three different coverages (5X, 10X and 20X) using the MASON read simulator [53] with the default error model. This led to 12 different sets of simulated data. L1 elements (L1 Homo sapiens, L1HS) were simulated with a similar strategy but only for heterozygous insertions using 106 bp reads at 10X and 20X coverage. One extra step in the L1 simulation was that simulated L1 elements were randomly truncated at 5′. The length distribution used for L1 truncation is derived from the L1 detection results in Stewart et al. 2011 (Figure 1A and 1B). All of the simulated reads had a 500 bp ± 100 bp (median ± standard deviation) insert size. MOSAIK 2.0 with default parameters was used to align these simulated reads against a customized human reference that combined hg19 and 23 ME sequences (4 Alu, 17 L1, 1 SVA and 1 HERV) downloaded from RepBase [47]. The output BAM files from MOSAIK were sorted by genomic coordinates using Bamtools [54]. The final BAM files served as the input to Tangram for MEI discovery and genotyping. RetroSeq calls were based on BWA [55] alignments with default parameters as suggested in the RetroSeq publication.

### Calculation of breakpoints in simulated data

Since the output format of Tangram is VCF, the reported breakpoints are in a 1-based system. The real breakpoint is determined as the last nucleotide before the inserted sequence. For events detected only by the RP signal, the confidence interval (left and right boundaries) around each breakpoint is calculated and reported in the final VCF in addition to the event location. For events with SR supporting fragments, we only report the breakpoint locations based on the left most convention because of the high resolution of the SR method.

### Genotype weighting for genotype accuracy estimation in simulated data for Alu

To estimate the genotype accuracy for each parameter set (read length and coverage) from the simulated data, we randomly chose 500 true positive MEI events reported by both Tangram and RetroSeq (Table 1). Of these, 400 were selected from the heterozygous simulation dataset, and 100 from the homozygous simulation dataset (the 4:1 ratio was based on experimentally validated genotypes from our earlier study, Stewart et al. 2011). The genotype accuracy was then calculated for these loci by comparing the designated genotype with the predicted genotype from the MEI detectors. The random selection and genotype accuracy experiment was then repeated five times (to give a sample of 2,500 MEI loci) and the overall genotype accuracy was determined by averaging the results of the five experiments (Table 2). Since for L1 simulation we only generated heterozygous datasets, there is no weighting step for genotype accuracy assessment.

### Identification of events across MEI callsets

In estimating the sensitivity of the call sets from Tangram, RetroSeq and TEA from the deep sequencing CEU trio data, an MEI event is deemed to match the locus in Stewart et al. 2011, if the two events are within 500 bp of each other. This criteria is a result of the large breakpoint uncertainty in Stewart et al. 2011. Also it is the 1000GP standard for validation experiments. We used the same window for consistency between comparisons to validation results and reference results from Stewart et al. 2011.

### Command line used for calling MEI with RetroSeq

We used following command lines to call MEIs on simulation dataset with RetroSeq:


### Software availability

The source code and documentation are available at https://github.com/jiantao/Tangram. Tangram is also available as part of our pipeline and tool launcher system, GKNO, which is available at https://github.com/gkno.

### PCR validation

Two sets of 80 loci each were selected for PCR validations from the whole dataset (detected with 23 1000GP phase 1 samples) of candidate loci containing Alu, L1, SVA, and LTR elements. The first set contained loci from the whole dataset while the second set included only loci identified as novel based on previous studies [27, 38–44] and the dbRIP database [45]. Due to the nature of paired-end reads and low coverage data, breakpoint coordinates for MEIs were commonly not available. Thus, an insertion range was provided for each locus within which the MEI was predicted. For primer design, 600 bp of flanking sequence were added upstream and downstream of the insertion coordinates. The sequence was extracted from the human reference genome (hg19) using Galaxy [56–58].

Alu elements were masked using RepeatMasker [51]. After adding a safety margin of 50 nucleotides up- and downstream of the insertion coordinates, primers were selected using BatchPrimer3 v2.0 [59]. The uniqueness of each primer was determined using BLAT [60]. An *in silico* PCR was performed for each locus when at least one primer had more than one match. If several matches were identified or the *in silico* PCR provided evidence for more than one PCR product primers were manually redesigned. In these cases the repeat content of the flanking sequence was determined using RepeatMasker. Moreover, the flanking sequence was ‘Blatted’ against the human reference genome (hg19) to determine if the flanking sequence matched to highly homologous loci. In cases with high sequence homology, the other orthologous sequences were retrieved using the UCSC genome browser [52]. Following a multiple alignment of the candidate locus with the other orthologous loci using BioEdit [61] primer design was attempted in regions with sequence divergence between the different loci. All manually designed primers were tested with Primer3 [62]. For loci with ambiguous PCR results, no amplification, or amplification of only the empty insertions site, a second primer pair was designed using the same primer design criteria described above.

Due to the size and high GC content of SVA elements, we used previously designed internal PCR primers [27]. The internal primers were designed within the 3′ end of the SVA sequence matching the consensus sequences of the youngest SVA subfamily (SVA_F), which is human-specific. All PCR primers were ordered from Sigma Aldrich, Inc. (St. Louis, MO). The PCR primer sequences used in this validation study are available at https://biosci-batzerlab.biology.lsu.edu/supplementary_data/BC_Tangram_MEI_ValidationPCRprimers.xlsx.

### Availability of supporting data

All sequencing data from 1000 Genomes Project are available at the following ftp sites:

1. EBI FTP: ftp://ftp.1000genomes.ebi.ac.uk/vol1/ftp/.
2. NCBI FTP: ftp://ftp-trace.ncbi.nih.gov/1000genomes/ftp/.

The PCR primer sequences used in the validation experiment is available at:

https://biosci-batzerlab.biology.lsu.edu/supplementary_data/BC_Tangram_MEI_ValidationPCRprimers.xlsx.

## Abbreviations

MEI: Mobile element insertion
RP: Read pair
SR: Split read
SV: Structural variation
SNP: Single nucleotide polymorphism
NGS: Next-generation sequencing
CNV: Copy number variation
LTR: Long terminal repeat
L1: Long interspersed element 1
ERV: Endogenous retroviruse
BAM: Binary alignment map
CPU: Central processing unit
VCF: Variant call format
TSD: Target site duplication
FDR: False discovery rate
AFS: Allele frequency spectrum
ASW: African ancestry in Southwest USA
LWK: Luhya in Webuye, Kenya
YRI: Yoruba in Ibadan, Nigeria
PCR: Polymerase chain reaction
API: Application Programming Interface
SSW: Single instruction multiple data Smith-Waterman.
